# Potential Intermediate Hosts for Coronavirus Transmission: No Evidence of Clade 2c Coronaviruses in Domestic Livestock from Ghana

**DOI:** 10.3390/tropicalmed4010034

**Published:** 2019-02-10

**Authors:** Philip El-Duah, Augustina Sylverken, Michael Owusu, Richmond Yeboah, Jones Lamptey, Yaw Oppong Frimpong, Vitus Burimuah, Christopher Antwi, Raphael Folitse, Olivia Agbenyega, Samuel Oppong, Yaw Adu-Sarkodie

**Affiliations:** 1 Department of Clinical Microbiology, Kwame Nkrumah University of Science and Technology, PMB, UPO, Kumasi 00233, Ghana; elduahphilip9@gmail.com (P.E.-D.); Yeboahrichmond82@yahoo.com (R.Y.); jlamptey80@gmail.com (J.L.); 2 Kumasi Centre for Collaborative Research in Tropical Medicine, PMB, UPO, Kumasi 00233, Ghana; annan@kccr.de (A.S.); owusumichael-gh@hotmail.com (M.O.); oppongfrimpong1@gmail.com (Y.O.F.); vitus7uk@yahoo.co.uk (V.B.); 3 Department of Theoretical and Applied Biology, Kwame Nkrumah University of Science and Technology, PMB, UPO, Kumasi 00233, Ghana; 4 Department of Medical Laboratory Technology, Kwame Nkrumah University of Science and Technology, PMB, UPO, Kumasi 00233, Ghana; 5 Department of Animal Science, Kwame Nkrumah University of Science and Technology, PMB, UPO, Kumasi 00233, Ghana; cantwi@icloud.com; 6 School of Veterinary Medicine, Kwame Nkrumah University of Science and Technology, PMB, UPO, Kumasi 00233, Ghana; raphfolitse@yahoo.com; 7 Department of Agroforestry, Kwame Nkrumah University of Science and Technology, PMB, UPO, Kumasi 00233, Ghana; olivia_agbenyega@yahoo.com; 8 Department of Wildlife and Range Management, Kwame Nkrumah University of Science and Technology, PMB, UPO, Kumasi 00233, Ghana; kobbyoppong@yahoo.com

**Keywords:** livestock, coronavirus, intermediate host, bats

## Abstract

The emergence of Middle East Respiratory Syndrome Coronavirus (MERS-CoV), nearly a decade ago with worldwide distribution, was believed to be of zoonotic origin from bats with dromedary camels as intermediate hosts. There is a likelihood of other domestic livestock serving as intermediate hosts for this virus. The presence of coronaviruses, closely related to MERS-CoV in Ghanaian bats, presented the opportunity to test the hypothesis of transmissibility of this virus through domestic livestock species. The possible interactions between livestock and bats in 31 household farms were accessed by observation and interviews with farmers. Rectal swabs and serum from cattle, sheep, goats, donkeys, and swine from commercial and household farms were tested for MERS-CoV and a *Nycteris* sp. bat coronavirus, previously detected in Ghana. A pan-PCR assay to detect clade 2c viruses and recombinant immunofluorescence assay to detect anti-spike IgG antibodies against the target viruses were used. Likely contact between livestock and bats was determined for 13 farms (41.9%) that reported confining their livestock and also observing bats in their homes. Livestock were left unconfined on eight farms (25.8%) that also observed bats roosting in trees close to their homes. No viral RNA or antibodies against the two coronaviruses were detected in any of the livestock species tested. Cattle, sheep, goats, donkeys, and swine are not likely hosts of clade 2c coronaviruses.

## 1. Introduction

Coronaviruses are enveloped viruses with positive-strand RNA genomes of size ranging from 26–32 kilobases that are pathogenic to both mammals and birds [1]. According to the International Committee on Taxonomy of Viruses, coronaviruses belong to the order *Nidovirales* and family Coronaviridae and are classified into four genera namely *Alphacoronavirus*, *Betacoronavirus*, *Gammacoronavirus,* and *Deltacoronavirus* [2]. Two novel betacoronaviruses that have emerged as human pathogens within the last twenty years, which have caused outbreaks with high case fatality proportions, are the previously unknown coronaviruses called severe acute respiratory syndrome coronavirus (SARS-CoV) and the Middle East respiratory syndrome coronavirus (MERS-CoV). SARS-CoV belongs to sub-group 2b of the genus *Betacoronavirus* and is identified as the cause of a severe respiratory disease that emerged and caused an international epidemic in 2002–2003 [1,3]. SARS-like coronaviruses have been found in Himalayan palm civets and humans in live animal markets in China [4] where the disease is believed to have originated due to close contact between bats, civets, and humans in the wildlife trade [5]. MERS-CoV is a member of sub-group 2c of the betacoronaviruses and was identified in patients with severe respiratory disease in the Middle East in 2012 [6]. Later, MERS-CoV sequences were also detected in nasal swabs of dromedary camels in the Middle East where the disease was predominant [7]. A further report documenting the transmission of MERS from camels to a human contact was subsequently made [8]. This supports the hypothesis that the disease was passed to humans from camels. MERS-CoV-like coronaviruses have been isolated from bats in the Middle East which indicates that bats may also play a role in human infections [9]. Bats have been identified as the source of most human coronaviruses, some of which are believed to have used livestock intermediate hosts for transmission to humans similar to MERS-CoV which made use of dromedary camels [9,10], SARS-CoV which made use of Himalayan palm civets (*Paguma larvata*) [4,11], and human coronavirus 229E (HCoV-229E) which made use of camelids [12,13,14]. Human coronavirus NL63, however, does not have any known intermediate host but viruses closely related to this have also been found in bats [15].

Contact with camels has been identified as one of the risk factors for contracting MERS [7,16]; however, some seropositive cases of MERS-CoV did not rear camels but rather other domestic livestock namely cattle, sheep, goats, and donkeys [17]. This raises the possibility of these other species being involved in the transmission of MERS-CoV. Several studies investigating MERS-CoV, particularly in the Arabian Peninsula, also sort to assess the possible role of these other livestock species in the transmission of MERS-CoV to humans but these studies generally employed small sample sizes [18,19,20,21] which may lack the power to detect this occurrence. Although there has been no autochthonous virus detection of MERS-CoV in Africa, the diversity of African viruses is larger than that in the Arabian Peninsula and suggests an African origin of MERS-CoV considering the documented trade of camels between the greater horn of Africa and the Arabian Peninsula [22]. 

A novel betacoronavirus also belonging to clade 2c and closely related to MERS-CoV was found to be circulating at a prevalence of 24.9% among *Nycteris* sp. bats in a study conducted in Ghana between 2009 and 2011 (BtCoV/KW2E-F93/Nyc_spec/GHA/2010) [23]. With interaction between bats, livestock, and humans, there is a tendency for humans to have been exposed to this virus either directly or with livestock as an intermediate host. Given the tendency for the use of intermediate hosts by the previously described betacoronaviruses prior to human infection, exposure of livestock species to this bat virus similar to MERS-CoV could be a significant step for the eventual spillover into the human population. Knowledge of circulating betacoronaviruses in the livestock population with a potential for emergence is important to help predict the next major coronavirus outbreak. The purpose of this study was to assess the potential of Ghanaian domestic livestock serving as intermediate hosts of clade 2c coronaviruses by serology and virologic detection with a relatively large number of livestock.

## 2. Materials and Methods 

### 2.1. Study Site Selection

A comprehensive list of livestock farms across the country was generated in consultation with various regional veterinary officers, and information on herd size and husbandry practices was also obtained. Some of these farms were shortlisted, and after confirmation of the information provided, shortlisted farms were then sensitized and informed consent was obtained before conducting the sampling.

### 2.2. Sample and Data Collection 

Rectal samples were collected from 35 farms, and serum samples were obtained from 24 of these 35 farms from June 2015 to May 2016. Information on livestock housing and bat locations was obtained from a total of 31 farms by observation and a questionnaire. This information was available for 22 of 24 farms from which serum samples were also obtained (Table 1). A total of 708 (16.6%) of the rectal samples and 133 (22.4%) of the serum samples were collected from farms without questionnaire data. Each animal was sampled once. 

**Figure 1:**
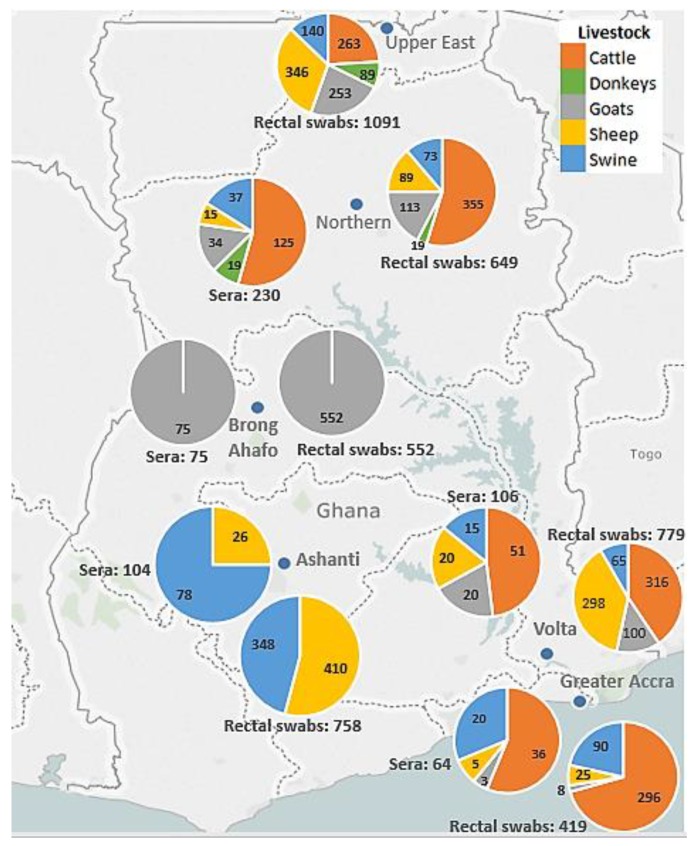
Map of Ghana showing distribution of livestock samples collected across the country.

A total of 4248 rectal swabs and 592 serum samples from cattle, sheep, goats, swine, and donkeys from the 35 farms located in the Ashanti, Brong Ahafo, Northern, Upper East, Volta, and Greater Accra regions of Ghana were collected in the study (Figure 1).

### 2.3. Ethical Issues

Permission for livestock sampling was obtained from the wildlife division of the Ghana Forestry Commission (Approval Number: AO4957, 28.04.2009).

### 2.4. Viral RNA Extraction

Viral RNA extraction from rectal swabs was performed using a slightly modified version of the Qiagen Viral RNA Mini Kit protocol (Qiagen GmbH, Hilden, Germany). Firstly, 200 μL of each sample in 800 μL of AVL lysis buffer (Qiagen GmbH, Hilden, Germany) was heat inactivated at 70 °C for 10 min. After pulse vortexing and short centrifugation, samples in pools of seven comprising 100 μL of each sample were extracted by the subsequent steps outlined in the Qiagen Viral RNA Mini Kit protocol and eluted in a final volume of 100 μL.

### 2.5. PCR for Clade 2c Coronaviruses

Virologic testing to generate amplicons of sub-group 2c betacoronaviruses for sequencing was done using a previously described PCR assay [24]. Briefly, two rounds of reverse transcriptase hemi-nested PCR were performed using the SuperScript III One-Step RT-PCR system and 10X *Taq* DNA polymerase PCR buffer (Invitrogen, Thermo Fisher Scientific, Waltham, MA, USA). This was followed by gel electrophoresis using a 2% agarose gel.

### 2.6. Recombinant Immunofluorescence Assay for Spike Protein of Clade 2c Coronaviruses 

**Figure 2:**
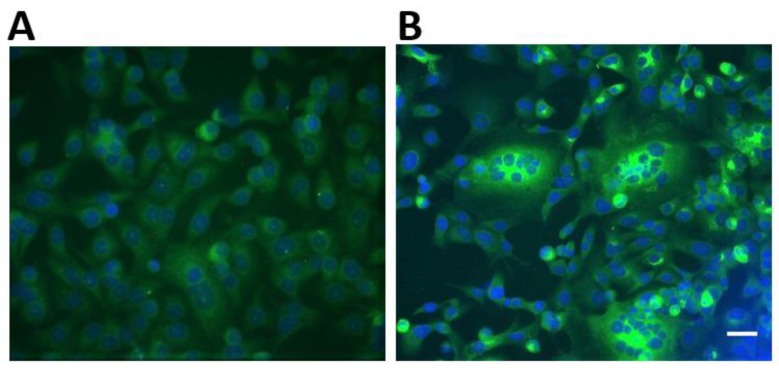
Recombinant immunofluorescence reactivity patterns of human MERS-CoV-positive and negative sera on Vero B4 cells used as a guide for the interpretation of livestock testing. (**A**) depicts a non-reactive sample characterized by low fluorescence intensity. (**B**) depicts a reactive sample showing high fluorescence intensity, particularly in cell syncytia. Scale bar indicates 20 μm.

Screening of livestock sera for immunoglobulin G (IgG) antibodies against MERS-CoV and the clade 2c coronavirus discovered in *Nycteris* sp. bats in Ghana was done using a previously described recombinant immunofluorescence assay [25]. Briefly, Vero B4 cells were co-transfected with pCG1 plasmids bearing the full spike proteins of the two coronaviruses of interest. After overnight transfection, the cells were fixed with ice-cold acetone/methanol (ratio 1:1). Sera to be tested were diluted 1:40 in a 1X concentration of a protein-free blocking solution (Roti^®^-Block, Carl Roth, Karlsruhe, Germany) and applied for 1 h at 37 °C. Secondary antibody detection was performed using Alexa488 fluorescent reporter-conjugated goat anti-bovine, goat anti-horse, goat anti-swine, donkey anti-sheep, and donkey anti-goat IgG antibodies (Dianova GmbH, Hamburg, Germany). The interpretation of results was determined as shown in Figure 2. All secondary antibodies used for the detection have been reported to work for the various species [20,26]. Livestock sera were inactivated at 56 °C for 30 min prior to testing.

### 2.7. Determination of Minimum Detectable Seroprevalence

In the event all the serum samples tested negative for IgG antibodies, an estimation of the minimum seroprevalence our sample of 592 could potentially detect in the total number of livestock sampled was determined as previously described [27,28]. Briefly, the relation used was:

*P_max_* = 1 − (1 − *P*_*x*>0_)^1/*n*^

where *Pmax* is the maximum seroprevalence in the sampled livestock, *n* is the number of serum samples tested from the sampled livestock and *P*_*x*>0_ is the desired probability of obtaining a seropositive sample which in this case was 95%.

## 3. Results

### 3.1. Locations of Livestock and Bats 

**Figure 3:**
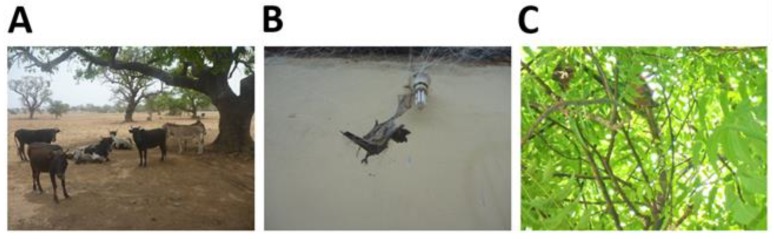
Free-roaming livestock under trees observed in a community in the Upper East region (**A**). A dead bat caught in a mist net close to the roof of a house in a farm in the Ashanti region (**B**). *Eidolon helvum* bats observed roosting in a tree at a farm site in the Volta region (**C**).

Farm owners and managers from 14 (45.2%) farms visited reported that they only kept their livestock confined in animal houses in close proximity to their homes and 2 (6.5%) only kept their livestock in animal houses approximately more than 10 m from their homes. Eleven farms (35.5%) did not keep their livestock in animal houses most of the time and were allowed to roam freely (Table 2, Figure 3A). Bats were reported by 16 (51.6%) farms to inhabit both their homes and trees close to their homes and were observed, particularly, in roofs and ceilings of houses (Table 2, Figure 3B). Bats were also reported to roost only in trees in the vicinity of farms (*n* = 7, 22.6%) as shown in Figure 3C but none of the farms visited reported knowledge of any bat caves in the vicinity of their farms (Table 2). 

### 3.2. Possible Contact between Bats and Livestock

**Figure 4:**
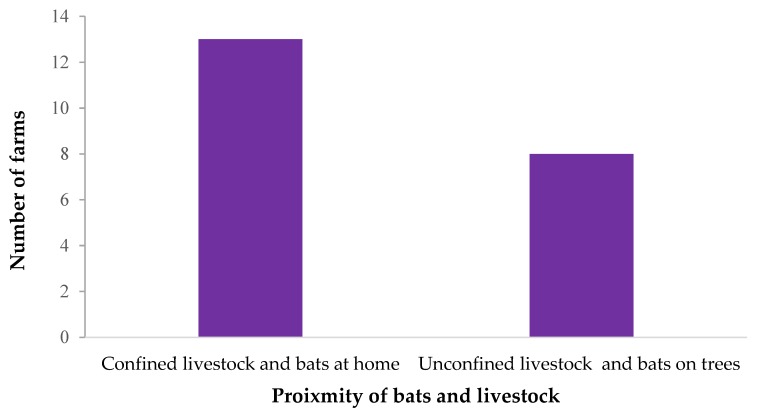
The frequency of situations likely to lead to contact between livestock and bats on farms visited.

More farms reported confining their livestock at home and also observing bats in their homes (*n* = 13, 41.9%) than leaving their animals unconfined and also observing bats in trees in the vicinity (*n* = 8, 25.8%), as shown in Figure 4. The proximity between livestock and bats is likely to present opportunities for contact that may lead to bat viruses potentially being introduced into livestock.

### 3.3. Laboratory Testing of Livestock Samples

No viral RNA of any clade 2c coronavirus was detected after PCR testing and Sanger sequencing of all bands of approximate expected sizes. All sera tested from the five species were negative for IgG antibodies against both MERS-CoV and the *Nycteris* sp. coronavirus, previously detected in Ghana.

### 3.4. Minimum Detectable Seroprevalence

With no detection of IgG antibodies in the 592 serum samples tested, this study determined with a 95% confidence that the seroprevalence of the tested clade 2c coronaviruses in the sampled livestock was less than 0.5%.

## 4. Discussion

Following the emergence of MERS-CoV and the subsequent outbreak in 2012 [6], several studies found that dromedary camels were the likely source of this infection [10,29]. MERS-CoV RNA and antibodies against the virus were found in camels from areas where the disease was predominant, and people had been in contact with the camels as well [8]. Sporadic cases of MERS in the Middle East were presumed to be zoonotic because of the high diversity of the viruses isolated from humans which were suggested to be as a result of independent cases of virus introduction into the human population from an animal source [18,30,31]. Small cohorts of other livestock species, including sheep, goats, and cattle from Europe, Northern Africa, and the Middle East have been subjected to serological screening for MERS with no positive cases detected [19,21,32]. Taken together, the results from the present study representing sub-Saharan Africa and the previous ones suggest that these livestock species do not readily serve as hosts for MERS-CoV. MERS-CoV makes use of dipeptidyl peptidase 4 receptor which is highly conserved in a lot of species and suggests, in theory, a potentially wide range of hosts [33,34], and some in vitro studies have also pointed to the susceptibility of species, other than bats and camels, to MERS-CoV [35,36]. Field data as stated previously has, however, so far shown the contrary, and another study that employed a bioassay showed that the replicative capacity of MERS-CoV in non-camelid livestock namely sheep, horses, and goats was limited [37]. 

Although contact may exist between bats and the domestic livestock other than camels, this may not be intensive enough to lead to high exposure rates or there could be the absence of other driving factors [38] that could promote a host-switching event for the *Nycteris* sp. coronavirus. In the present study, contact with all bats was considered, and at that level, the prevalence of the *Nycteris* sp. bat coronavirus would be 1% as against 24.9% in the *Nycteris* sp. bats only [23]. This makes the likelihood of exposure of livestock to this particular virus relatively low. A high prevalence of potential zoonotic pathogens, as well as sustained close contact, is, however, important in increasing the likelihood of transmission [39]. Although the potential for contact between bats and livestock was established in this study, prolonged close contact was not conclusively determined and may not prevail.

The low limit of detectable seroprevalence determined in this study is also another factor that may contribute to the absence of seropositive samples. With the shorter lifespan of RNA post infection compared to serum IgG [40], the likelihood of finding viral RNA among the sampled livestock would likely be much lower given the very low expected prevalence even for IgG. 

Unless the clade 2c coronaviruses adapt to livestock and persist after host switching, this is expected to be a rare occurrence, and the cohort of samples taken in the study could miss this as the sampling was done in the duration of a year and markers of infection are not permanent. In this scenario, an isolated situation of bat viruses jumping to livestock many years prior to the study would not likely be found.

In the event of fairly sustained contact between bats and livestock, additional barriers namely overcoming the host immune system or lack of an appropriate host receptor or entry-mediating endogenous proteases may prevent transmission all together [34]. This virus shares some similarities with the MERS-CoV and, as such, may not possess the ability to infect and replicate in most of the domestic livestock species tested, as has been demonstrated with MERS-CoV [18,19,21,23]. There are no known indigenous camelids among Ghanaian wildlife or kept on a large scale as livestock [41]. Given that these are known important hosts of coronavirus transmission [8,13], their introduction into Ghana on a large enough scale could bridge the gap between bats and humans and facilitate the possible transmission of some of the highly diverse viruses that circulate in the local bat population like the *Nycteris* sp. coronavirus to humans. 

Consumption of bats as bushmeat, which is prevalent in Ghana [42], is an important risk factor that could facilitate a host switching event directly to humans, and a serological survey of humans with high exposure to *Nycteris* sp. bats for antibodies against this virus may shed some light on potential transmission. 

## Figures and Tables

**Table 1:** Distribution of livestock samples from study sites.

Farms *n* = 35	Number of Rectal Samples per Farm (Percentage) *n* = 4248	Number of Serum Samples per Farm (Percentage) *n* = 592
AC	100 (2.4)	20 (3.4)
AF	77 (1.8)	2 (0.3)
AGD	618 (14.5)	61 (10.3)
AGV	132 (3.1)	30 (5.1)
AGB	52 (1.2)	-
AGO	134 (3.2)	-
AKA	24 (0.6)	2 (0.3)
AKI	70 (1.6)	5 (0.8)
AKU	148 (3.5)	14 (2.4)
AA	284 (6.7)	56 (9.5)
AI	193 (4.5)	108 (18.2)
ANY **	70 (1.6)	-
ATY	108 (2.5)	-
ATA	85 (2)	-
AW	1 (0)	-
AZ	81 (1.9)	-
ESBS	410 (9.7)	26 (4.4)
IB	50 (1.2)	9 (1.5)
JO	99 (2.3)	10 (1.7)
KB	33 (0.8)	19 (3.2)
KGBS **	552 (13)	75 (12.7)
KAS **	58 (1.4)	58 (9.8)
KT	51 (1.2)	-
KF	290 (6.8)	20 (3.4)
LB	137 (3.2)	-
LF	40 (0.9)	10 (1.7)
MS **	28 (0.7)	-
NS	108 (2.5)	-
NF	26 (0.6)	26 (4.4)
PT	65 (1.5)	4 (0.7)
SY	23 (0.5)	3 (0.5)
TH	16 (0.4)	15 (2.5)
WF	45 (1.1)	10 (1.7)
YA	17 (0.4)	3 (0.5)
ZM	23 (0.5)	6 (1)

N: Total number of samples collected. ** No data on bat proximity to livestock.

**Table 2:** Reported locations of livestock and bats at farms visited.

Animal	Location	Reported on the farm (N = 31)
		*n* (%)
Livestock	Only confined close to home (<10 m)	14 (45.2)
	Only confined away from home (>10 m)	2 (6.5)
	Confined both close and away from home Unconfined	4 (12.9) 11 (35.5)
Bats	At home only	0 (0)
	In trees close to farm only At home and in trees close to the farm Neither at home or in trees close to the farm	7 (22.6) 16 (51.6) 8 (25.8)
	In bat caves	0 (0)

N = Number of farms visited, *n* = Number of farms reporting situation, % = Percentage, m = Meters. NB: Percentages may not sum up exactly to 100 due to rounding.
